# Koebner Phenomenon Induced by Eyeglasses in a Patient With Discoid Lupus Erythematosus

**DOI:** 10.1155/crdm/8262393

**Published:** 2025-10-16

**Authors:** Sam Fathizadeh, Alexander D. Woods, Roger Haber

**Affiliations:** ^1^College of Medicine, University of Illinois Chicago, Chicago, Illinois, USA; ^2^Department of Dermatology, University of Illinois Chicago, Chicago, Illinois, USA

**Keywords:** chronic cutaneous lupus erythematosus, discoid lupus erythematous, Koebner phenomenon, lupus, systemic lupus erythematosus

## Abstract

The Koebner phenomenon, or isomorphic response, refers to the induction of disease-specific lesions on uninvolved skin following trauma. This phenomenon is well-documented across several dermatologic conditions such as psoriasis and vitiligo but is less recognized in discoid lupus erythematosus (DLE). We report a case of a 37-year-old African American woman with DLE, triggered by repeated contact with eyeglass frames, leading to the development of discoid lesions at the points of pressure on her face. This case is a rare occurrence of Koebnerization in DLE, diagnosed through characteristic histopathological findings and supported by serological evidence. Her management included hydroxychloroquine, topical corticosteroids, and methotrexate due to disease flaring. Eventually, she developed systemic lupus erythematosus, complicating her clinical course. This case discusses the need for awareness regarding Koebnerization in DLE and its potential role in indicating active disease or predicting transition to systemic involvement. Further research is necessary to explore the pathogenic mechanisms and implications of Koebnerization in DLE, emphasizing the importance of preventive measures against mechanical trauma in susceptible individuals.

## 1. Introduction

The Koebner phenomenon, or the isomorphic response, refers to the appearance of disease-specific lesions on previously unaffected skin following trauma [1]. It is well-documented in dermatology, including psoriasis, vitiligo, and lichen planus [1, 2]. According to Boyd and Neldner, its presence in other diseases, such as discoid lupus erythematosus (DLE), is considered occasional [3]. We describe a patient with Koebnerizing DLE, where trauma from eyeglasses induced discoid lesions on the face.

## 2. Case Report

**Figure 1:**
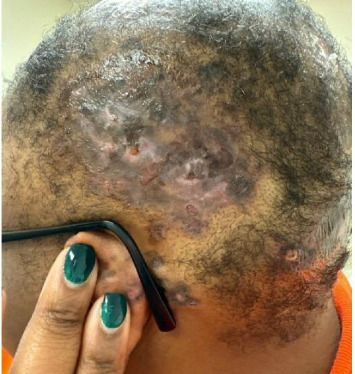
Scalp showing multiple areas of hair loss with violaceous, hyperkeratotic plaques exhibiting ulceration and scarring.

**Figure 2:**
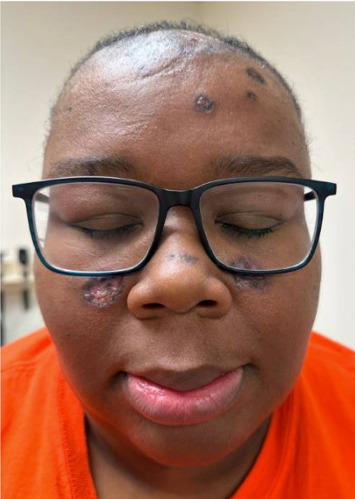
Recently developed round pink to purple plaques on the right and left cheeks, observed at the points of contact with the patient's glasses.

A 37-year-old African American woman presented with a 5-year history of discoid lesions on her scalp and arm. Physical examination of the scalp revealed alopecic violaceous hyperkeratotic plaques with ulceration and scarring (Figure 1). Recently, round plaques were observed on bilateral cheeks, nose, and retroauricular areas at the contact points of her glasses (Figure 2). She denied systemic symptoms. Her initial laboratory results revealed positive anti-Smith/RNP antibodies and speckled antinuclear antibody (ANA) cytoplasmic pattern at a titer of 1:160, but negative anti-Smith and anti-dsDNA antibodies. Punch biopsies of the arm and scalp revealed vacuolar interface change with dyskeratotic keratinocytes, with underlying lichenoid infiltrate composed of lymphocytes, histiocytes, and rare eosinophils, and increased dermal mucin, and IgG, IgM, IgA, C3, and fibrinogen deposition along the dermoepidermal junction, consistent with DLE, without evidence of systemic lupus erythematosus (SLE).

Hydroxychloroquine 200 mg twice daily and topical corticosteroids were initiated at her initial dermatology visit. Clobetasol propionate 0.05% ointment was applied to the scalp twice daily and fluocinonide ointment to facial lesions twice daily, with counseling on steroid side effects and site-specific application. After 6 months, due to disease flaring, methotrexate 15 mg weekly was added while hydroxychloroquine was maintained, along with daily folic acid supplementation except on methotrexate days. Intralesional triamcinolone acetonide (20 mg/mL) was administered to scalp lesions at four-week intervals.

The patient subsequently developed SLE, confirmed by positive dsDNA (120, normal < 24) and anti-Smith antibodies (59, normal < 40), elevated ESR (77, normal < 20), and proteinuria (100 mg/dL). A 24 h urine protein measurement was not performed, so the degree of proteinuria could not be expressed in mg/24 h. No renal biopsy was performed given the absence of additional clinical or laboratory findings suggestive of proliferative lupus nephritis requiring histologic confirmation.

## 3. Discussion

The Koebner phenomenon has been described in autoimmune conditions, though its association with DLE is rare [4–6]. DLE characteristically affects the face, scalp, and upper trunk, so the facial and retroauricular lesions in this patient are consistent with its typical distribution [7]. In lupus, the Koebner phenomenon is rare, often partial, and usually reflects active or uncontrolled disease rather than serving as a primary pathogenic driver [8]. Triggers include environmental stresses such as scratches, sun exposure, scars, tattoos, and piercings [6, 9]. The pathogenesis is theorized to have an initial nonspecific inflammatory step that releases cytokines, stress proteins, adhesion molecules, or autoantigens, followed by a disease-specific immune response involving T-cells, B-cells, autoantibodies, and immune deposits [6].

Koebnerization has been reported in DLE and dermatomyositis [2, 6]. In both, trauma-induced activation of keratinocytes and immune cells release cytokines and chemokines, which promote inflammation and lesion formation [6]. Elevated interferon pathways contribute to the pathogenesis of dermatomyositis and DLE. In DLE, interferon pathways play a significant role through plasmacytoid dendritic cells and keratinocytes, which establish a self-amplifying inflammatory loop driven by Type I interferons [2]. The presence of Koebnerization in both dermatomyositis and DLE suggests that trauma-induced activation of interferon pathways might be a common pathogenic mechanism in these conditions, contributing to the chronic inflammatory lesions observed in both diseases.

While there are reports of the Koebner phenomenon following a tattoo, there are no documented cases of repeated microtrauma from eyewear in patients with DLE [5]. Our patient developed symmetric discoid lesions at the points of contact with her eyeglasses on her cheeks and nose. The chronic friction and pressure from the eyeglass frames likely induced these DLE lesions. While in other conditions, such as vitiligo, Koebnerization is associated with active disease and risk for rapidly progressive disease; it is not well elucidated if this portends a similar disease state in DLE. Further research is necessary to determine if Koebnerization is a marker of active DLE and whether it portends a higher risk for SLE, as in our patient. A variety of traumatic or scar-related events have been reported to precipitate new lesions in DLE, and Table 1 summarizes representative literature cases along with the current case to illustrate the range of potential triggers and clinical presentations.

## 4. Conclusion

Koebnerization may occur during active DLE. In this case, whether clinicians recognized the phenomenon or not, it would not have changed management for a patient presenting with disfiguring DLE of the face and scalp. When possible, traumatic factors should be minimized or withdrawn, but some lesions will inevitably arise. Education is important because Koebnerization can manifest after a delay. Further research is needed to understand the implications of developing the Koebner phenomenon in DLE and its potential role in indicating disease activity and risk for SLE, as well as, to guide management.

## Figures and Tables

**Table 1:** Selected reported trauma- and scar-associated lesions in discoid lupus erythematosus, including the present case.

Trauma/trigger	Location	Lesion type	Author & year
Scratch	Face/scalp	Discoid plaque	Dobrzyńska et al. [4], 2021
Sunburn	Face	Discoid plaque	Ueki [8], 2005
Scar	Various	Discoid plaque	Ueki [8], 2005
Tattoo	Forearm	Discoid plaque	Kluger et al. [9], 2021
Piercing	Ear	Discoid plaque	Kluger et al. [9], 2021
Heat (lupus ab-igne)	Back	Discoid plaque	Berger et al. [5], 2012
Eyewear pressure	Cheeks, retroauricular areas	Discoid plaque	Present case

## Data Availability

The data that support the findings of this study are available from the corresponding author upon reasonable request.

## Nomenclature

DLE: Discoid lupus erythematosus
SLE: Systemic lupus erythematosus
ANA: Antinuclear antibody
